# Recent progress in West Nile virus diagnosis and vaccination

**DOI:** 10.1186/1297-9716-43-16

**Published:** 2012-03-01

**Authors:** Marina De Filette, Sebastian Ulbert, Mike Diamond, Niek N Sanders

**Affiliations:** 1 Laboratory of Gene Therapy, Faculty of Veterinary Sciences, Ghent University, Heidestraat 19, 9820 Merelbeke, Belgium; 2 Vaccine Technologies Unit, Fraunhofer Institute for Cell Therapy and Immunology, Perlickstrasse 1, D-04103 Leipzig, Germany; 3 Departments of Medicine, Molecular Microbiology and Pathology & Immunology, Washington University School of Medicine, 660 South Euclid Avenue, Box 8051, St. Louis, Missouri 63110, USA

## Abstract

West Nile virus (WNV) is a positive-stranded RNA virus belonging to the Flaviviridae family, a large family with 3 main genera (flavivirus, hepacivirus and pestivirus). Among these viruses, there are several globally relevant human pathogens including the mosquito-borne dengue virus (DENV), yellow fever virus (YFV), Japanese encephalitis virus (JEV) and West Nile virus (WNV), as well as tick-borne viruses such as tick-borne encephalitis virus (TBEV). Since the mid-1990s, outbreaks of WN fever and encephalitis have occurred throughout the world and WNV is now endemic in Africa, Asia, Australia, the Middle East, Europe and the Unites States. This review describes the molecular virology, epidemiology, pathogenesis, and highlights recent progress regarding diagnosis and vaccination against WNV infections.

## Table of contents

1. Introduction

2. West Nile virus

3. Epidemiology

4. Pathogenesis

5. Diagnosis

5.1. Nucleic acid based tests for WNV

5.2. Serologic diagnosis of WNV infections

5.3. WNV antigen detection

6. Vaccination

6.1. Licensed West Nile virus vaccines for animals

6.2. WNV vaccines under development

6.3. Clinical trials with West Nile virus vaccines in humans

7. Conclusions

8. Competing interests

9. Authors' contributions

10. Acknowledgments

11. References

## 1. Introduction

West Nile virus (WNV) is a zoonotic mosquito-transmitted arbovirus belonging to the genus *Flavivirus *in the family *Flaviviridae*. WNV is maintained in a mosquito-bird-mosquito transmission cycle [1], whereas humans and horses are considered dead-end hosts. WNV is transmitted primarily by the bite of infected mosquitoes, themselves acquiring the virus by feeding on infected birds.

The West Nile virus has been reported in dead or dying birds of at least 326 species [2]. The clinical outcome of infection is variable e.g. chickens and turkeys are resistant to disease while some species are particularly susceptible, e.g. crows, Carolina chickadees, tufted titmice, blue jays, American robins, and eastern bluebirds.

WNV has a wide geographical range that includes portions of Europe, Asia, Africa, Australia (Kunjin virus) and North, Central and South America [3]. Migratory birds are thought to be primarily responsible for virus dispersal, including reintroduction of WNV from endemic areas into regions that experience sporadic outbreaks [3].

In humans, it was first isolated in the West Nile province of Uganda in 1937 from the blood of a woman suffering from a mild febrile illness [4]. Until the mid 1990's, West Nile (WN) disease was considered as a minor risk for humans and horses because it only appeared sporadically. The first cases of West Nile virus in its lethal encephalitic form were reported in Algeria in 1994. Since the first large outbreak in Romania in 1996, which was characterized by a high number of neuroinvasive cases, and the huge epidemics and equine epizootics into North America that followed West Nile virus introduction in New York city in 1999 [5], WN associated disease has become a major public health and veterinary concern.

## 2. West Nile virus

**Figure 1:**
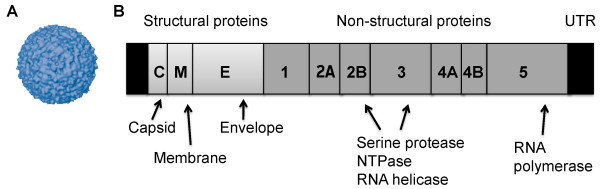
**The structure (a) and 11 kb long RNA genome (b) of West Nile virus**.

The structure of WNV particles have been elucidated by Mukhopadhay et al. [6]. Electron microscopy and image reconstruction techniques established that WNV is a small spherical icosahedral virus with a 50 nm diameter and a lipid envelope surrounding an icosahedral nucleocapsid consisting of capsid proteins that are associated with the RNA genome (Figure 1a). The sequence of the WNV genome and the function of the different viral proteins in the viral lifecycle as well as immune evasion have been described in detail elsewhere (Figure 1b) (reviewed by Ulbert et al. [7]).

**Figure 2:**
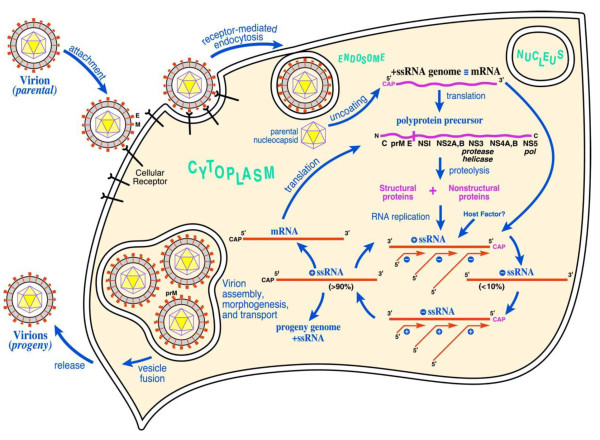
**West Nile virus life cycle**. After binding and uptake, the virion envelope fuses with cellular membranes, followed by uncoating of the nucleocapsid and release of the RNA genome into the cytoplasm. The viral genome serves as messenger RNA (mRNA) for translation of all viral proteins and as template during RNA replication. Copies are subsequently packaged within new virus particles which are transported in vesicles to the cell membrane. Reprinted with permission from PNAS 2002, vol. 99 no. 18 11555-11557. Copyright 2002 National Academy of Sciences, U.S.A.

During viral entry (Figure 2), the E protein interacts with one or more cell surface receptor(s). It is not completely clear which cellular receptors are involved in WNV binding, however DC-SIGN, alphaVbeta3 integrin [8] and laminin-binding protein [9] have been reported as potential receptors. After binding to the cell, the virus is taken up via clathrin-mediated endocytosis [10] and in the acidified endosome the E protein undergoes conformational changes resulting in fusion between the viral and cellular membranes [11]. After the fusion event the positive-stranded RNA genome is released into the cytoplasm of the cell. The viral RNA is translated into a single polyprotein [12], which is proteolytically processed to yield three structural proteins (the envelope protein E; the membrane precursor protein prM; and the capsid protein C) and seven non-structural (NS) proteins (NS1, NS2a, NS2b, NS3, NS4a, NS4b, and NS5). Whereas the cleavages at the junctions C-prM, prM-E, E-NS1, NS4A-NS4B [13], and likely also NS1-NS2A [14], are performed by the host signal peptidase located within the lumen of the ER, the remaining peptide bonds are cleaved by the virus encoded NS3 protease. *Flaviviruses *replication requires the viral protein NS5, which is an RNA-dependent RNA polymerase [15,16]. An "antisense" negative strand RNA is produced by this enzyme, which then serves as a template for the synthesis of many new copies of the infectious positive strand RNA genome.

WNV assembles on virus-induced membranes derived from the endoplasmic reticulum and buds into the lumen as immature virions on which E and prM proteins form 60 heterotrimeric spikes. Transit of the immature virion through the mildly acidic compartments of the trans-Golgi network triggers a rearrangement of E proteins on the immature virion; the lower pH induces a structural transition such that E proteins lie flat as antiparallel dimers on the surface of the virion [17]. Under acidic conditions, prM remains associated with the virion and protrudes from the surface of an otherwise smooth virus particle. This pH-dependent conformational change increases the susceptibility of prM for a furin-like serine protease. Cleavage and release of prM completes the virion maturation process, and is a required step in the virus lifecycle.

Flavivirus nonstructural (NS) proteins modulate the host antiviral response. This was recently reviewed by Diamond et al. [18]. Flavivirus NS1 is a versatile nonstructural glycoprotein, with intracellular NS1 functioning as an essential cofactor for viral replication [19] and antagonist of TLR signaling [20], whereas NS1 at the cell surface and secreted NS1 antagonize complement activation [21,22]. NS2A is involved in the biogenesis of virus-induced membranes, which have a vital role in virus assembly [23]. NS2A also inhibits interferon-β promoter activation [24]. The flavivirus non-structural protein NS4B and NS5 block activation of the JAK-STAT1 signaling pathway [25], which limits production of antiviral interferon stimulated genes in infected cells.

## 3. Epidemiology

Birds are the primary vertebrate hosts of WNV and in endemic regions, the virus is maintained in an enzootic cycle between *Culex *mosquitoes and birds [1]. Mosquito species from other genera are also susceptible to infection. There is now indirect evidence that WNV is transported by migratory birds to the temperate areas of Europe during spring migration [26-28]. Mammals are less important than birds in maintaining transmission cycles of the virus as viremia is too low in most of the mammal species to reinfect mosquitoes. Humans are regarded as dead-end hosts because the concentration of virus within the blood is insufficient to infect a feeding naïve mosquito [29]. However, in humans, the virus can spread between individuals by blood transfusion and organ transplantation [30]. Few reports describe the possible transmission from a mother to her newborn via the intrauterine [31] route or via breast-feeding [32].

West Nile virus was first isolated in Uganda in 1937 from the blood of a febrile woman [4]. It is now recognized as the most widespread of the flaviviruses, with geographic distribution in the United States of America, Australia, Africa and Eurasia. The largest outbreak of WNV in Europe to date was in Romania in 1996 when 800 clinical cases of neuroinvasive disease were reported and in 393 cases the presence of WNV was confirmed [33]. A total of 17 deaths were reported during this outbreak. Between 1997 and 2010 in Europe, WNV infections have been observed sporadically in Portugal, Spain, France, Czech Republic, Hungary and Italy and during a severe outbreak in Russia in 1999 (318 cases) [34]. However in 2010 the epidemiological situation changed with the second largest outbreak of the disease in the EU occurring in Greece [35]. During this outbreak 262 cases were confirmed with 197 patients suffering from West Nile neuroinvasive disease and 33 reported fatalities. In the same year 480 cases of West Nile infections were detected in Russia and also some cases were reported in Romania.

In 1999, WNV was detected for the first time in the Western Hemisphere in New York [36]. During this outbreak 62 human cases of WNV including 7 deaths were identified in New York. Despite intense mosquito control measures to minimize human infections, WNV spread into Canada and the remainder of the lower 48 continental states. WNV became endemic within 10 years of its introduction in North America. So far, in the United States between 1999 and 2010, 30 662 cases were confirmed and associated with 1163 deaths [37].

It has been recently proposed that WNV can be grouped into 7 lineages [38]. Two major genetic lineages of WNV have been well described based on phylogenetic analysis [39]. Lineage 1 is widespread and contains isolates from Europe, the USA, the Middle East, India, Africa and Australia. Lineage 1 is further segregated into three different clades: 1a, 1b and 1c. WNV-1a is mainly found in Europe, North America, Middle East and Africa. This clade can further be divided in six clusters with distinct evolutionary histories [40]. WNV-1b contains the Australian Kunjin virus and lineage 1c some Indian isolates. Lineage 2 contains isolates from Southern Africa and Madagascar. Since 2004 lineage 2 has also been observed in central [41] and Eastern [42] Europe. In 2010 it caused outbreaks in Romania [43] and Greece [44] and in 2011 it was detected for the first time in Italy [45]. The Greek and Italian strains showed the highest homology to Hungarian and South African strains, differing from the Russian lineage 2 strains. This means that at least two lineage 2 strains are circulating in Europe causing severe disease in humans. In general the lineage 1 viruses are considered to be more virulent than the lineage 2 viruses. However, animal experiments have demonstrated that highly and less neuroinvasive phenotypes exist in both lineages. Mutations responsible for increasing virulence in lineage 2 viruses have previously been described in lineage 1 viruses like the substitution of amino acids at position 249 for proline in NS3 [46,47]. Also the glycosylation state of the E protein is an important determinant of pathogenicity [48,49]. Many of the WNV strains responsible for more severe outbreaks of WN disease are glycosylated at position 154. The lineage 3 is represented by a virus strain that was isolated from mosquitoes in the Czech Republic, designated the Rabensburg virus [50] and lineage 4 was isolated from a thick from the Caucasus [51]. WNV strains from India belonging to a subcluster of lineage 1 are sometimes classified as lineage 5 [52]. The Sarawak Kunjin virus strain is significantly different to the other Kunjin viruses, and therefore re-classification of this virus as lineage 6 has been proposed. Furthermore, the African virus, Koutango, is closely related to the WN virus lineages, and could be considered as a seventh lineage.

## 4. Pathogenesis

Mosquitoes become infected with WNV after biting a bird with high-level viremia and may then transmit it to humans following a blood meal from the host. The human incubation period of West Nile virus is 2 to 14 days [53]. The majority (~75 to 80%) of humans infected with WNV usually have no or very mild symptoms. Approximately 20% of infected patients develop a febrile illness ("West Nile fever") with malaise, myalgias, headache and lymphadenopathy [54]. A small number of the symptomatic cases progress to the neuroinvasive form of WNV infection [55], which can be characterized by acute flaccid paralysis, meningitis, encephalitis and ocular manifestations. Overall, only 1 in 150 infections results in the most severe and potentially lethal form of the disease, although the relative risk is increased in the elderly or immunocompromised. Long-term complications (1 year or more after infection) are common in patients recovering from severe WNV infection. Since no specific therapy has yet been approved for humans, patients infected with WNV have limited treatment options. The primary course of action is supportive. Small numbers of patients have received antibody therapy against WNV infection [56] or off-label treatment with IFN-α [57].

In mammals, the initial replication of WNV after mosquito inoculation is believed to occur in Langerhans dendritic cells. These infected cells migrate to draining lymph nodes resulting in a primary viremia [58] and a subsequent infection of peripheral tissues such as the spleen and kidney. Viremia ensues and after spread to the visceral organs, WNV may cross the blood-brain barrier (BBB) and enter the central nervous system (CNS). The mechanism by which WNV cross the BBB remains largely unknown, although tumor necrosis factor alpha (TNF-α)-mediated changes in endothelial cell permeability have been proposed to facilitate CNS entry [59]. Other models have been proposed like infection of olfactory neurons and spread to the olfactory bulb [60], direct axonal retrograde transport from infected peripheral neurons [61] or transport of the virus by infected immune cells trafficking to the CNS [62]. Neuronal infection is associated with degeneration, a loss of cell architecture, and cell death. Later in the course of infection, a mononuclear cell infiltrate appears diffusely throughout infected regions, although it is not clear whether these inflammatory cells eradicate infection or contribute to pathogenesis by destroying infected neurons and releasing pro-inflammatory cytokines [63].

The protective immune response to WNV requires both innate and adaptive immunity. IFN-α and IFN-β are produced during the earliest stages of WNV infection after host cell recognition of viral RNA. Mice with a defect in type I IFN signaling are much more sensitive to WNV infection than their wild-type counterparts [64]. The complement also is required for protection from lethal WNV infection in mice. WNV activates complement in vivo, and mice lacking the central complement protein C3 showed enhanced lethality after WNV infection [65]. Macrophage uptake of WNV can control infection through direct viral clearance, enhanced antigen presentation, and cytokine and chemokine secretion [66]. γδ T cells also directly limit WNV infection in early immune responses [67]. As they lack classical MHC restriction, they can react with viral antigens in the absence of conventional antigen processing [68]. Furthermore, the importance of adaptive immunity has also been demonstrated as passive transfer of immune monoclonal and polyclonal antibodies protected mice from lethal WNV infection [69]. IgM is critically important for the control of early WNV infection and is detectable approximately 4 to 7 days after infection [70]. After four to five days of illness, IgG antibodies are measurable in patients presumably conferring long-term protection against WNV re-infection [71]. Cellular immune responses also control WNV infections. Cytolytic T cells clear WNV infection by lysing infected cells directly through the delivery of perforin and granzymes A and B [68,72]. CD8+ deficient mice develop persistent WNV infections in the brain and humans with impaired T cell function are at increased risk of neuroinvasive WNV infection and poor outcome [73].

## 5. Diagnosis

A review of the developments in WNV diagnosis was published in 2007 by Dauphin et al. [74]. The current paper focuses more on the new trends in diagnosis since 2007.

### 5.1. Nucleic acid based tests for WNV

Because the virus is present at very low levels in human blood and tissues, an in vitro amplification of the genetic material is used to enhance the detection rate of WNV infections. Several investigators have reported real-time PCR-based detection systems for rapid detection of WNV infection in clinical samples (Table 1).These real-time PCR systems rely upon the detection and quantification of a fluorescent reporter. Single-tube, real-time RT-PCR shows many advantages over end-point RT-PCR because it is more rapid, often more sensitive, more specific, and minimizes contamination. In addition, real-time RT-PCR is easily standardized and enable nucleic acid quantification. In the simplest and least expensive system the reporter is the double-strand DNA-specific dye SYBR Green. A disadvantage is that SYBR Green will bind to any double-stranded DNA in the reaction, including non-specific PCR products and primer-dimers. Papin et al. developed a SYBR Green based assay [75] that could detect 100% of the different WNV target region variants in their study, whereas a TaqMan assay failed to detect 47% of possible single nucleotide variations in the probe-binding site. Johnson et al. designed a pan-flavivirus RT-PCR utilizing degenerate primers targeting the NS5 gene to allow the detection of a range of flaviviruses including WNV. This SYBR Green based RT-PCR was able to detect WNV however the sensitivity was much lower compared to WNV-specific TaqMan RT-PCR assays [76]. SYBR Green has been shown to inhibit the PCR reaction to some extent and melt curve analysis is complicated by dye redistribution during melting. Eischeid analyzed the behavior of other DNA dyes in real-time PCR and found that EvaGreen and SYTO dyes 13, 16, 80 and 82 outperformed SYBR Green in real-time PCR [77].

**Table 1:** Overview of nucleic acid based assays for WNV detection.

Technique used	Reference
SYBR Green RT-PCR	[75,76]

TaqMan RT-PCR	[78]

TaqMan-MGB RT-PCR	[79]

Multiplex RT-PCR	[80,81]

Molecular beacon RT-PCR	[82]

RT-PCR/ESI-MS	[83]

RT-PCR/LDR	[84]

RT-PCR/FRET	[85]

RT-LAMP	[86]

NASBA	[87]

Digital PCR	Invitrogen

The two most popular alternatives to SYBR Green are TaqMan and molecular beacons, both of which use hybridization probes and rely on fluorescence resonance energy transfer (FRET) for quantification. A TaqMan qRT-PCR for rapid detection of WNV in human clinical specimens was first developed by Lanciotti et al. [78]. Compared to the traditional RT-PCR assay, this TaqMan RT-PCR was more sensitive and could detect less than 1 PFU of virus whereas the traditional RT-PCR had a detection limit of 1 PFU of virus [78]. Chao et al. developed a multiplex TaqMan RT-PCR for the simultaneous identification of four different flaviviruses [80], including YFV, JEV, WNV and SLEV with a detection limit of respectively 3.5, 2, 10 and 10 PFU/mL. Dyer et al. also developed a multiplexed TaqMan assay for the detection of SLE, WNV, Dengue and TBE species [88]. Naze et al. developed a multiplex real-time RT-PCR assay for the simultaneous detection and quantification of dengue virus (DENV) and Chikungunya virus (CHIKV) from plasma samples [81]. In parallel, a real-time RT-PCR assay was developed for the detection and quantification of WNV using the same blood extract and identical amplification conditions as for DENV and CHIKV [81]. Jiménez-Clavero et al. developed an improved TaqMan real-time RT-PCR by introducing a minor groove binder (MGB), a 3'-labeling group that in addition to acting as a quencher also increases the binding affinity between the probe and its target sequence [79]. Two different probes were used and all samples were analyzed in parallel with the TaqMan RT-PCR described by Lanciotti et al. Both methods performed comparably in terms of sensitivity and were able to detect all clade 1a isolates, however Kunjin and lineage 2 isolates were only detected by the new TaqMan-MGB real-time PCR.

Molecular beacons, like TaqMan probes, also contain fluorescent and quenching dyes but FRET only occurs when the quenching dye is directly adjacent to the fluorescent dye. Lee et al. developed a combined RT-PCR, using a FAM-labeled molecular beacon probe for WNV detection and a VIC-labeled TaqMan probe for internal control detection [82]. The assay was highly specific for WNV and demonstrated no reactivity with 15 other viruses.

Grant-Klein et al. combined RT-PCR with electrospray ionization mass spectrometry (ESI-MS) to detect tick- and mosquito-born flaviviruses on the Ibis T5000 platform [83]. The Ibis T5000 analyses DNA and determines the base composition (AxGxTxCx) of PCR amplicons by using ESI-MS. For the RT-PCR assay they developed eight primer pairs able to detect a broad range of flaviviruses. WNV was correctly detected by RT-PCR/ESI-MS in blood, serum and urine spiked with WNV demonstrating that these clinical matrices did not inhibit the detection of this virus. Using WNV, the sensitivity of the assay was determined to be approximately 2 PFU/mL.

**Figure 3:**
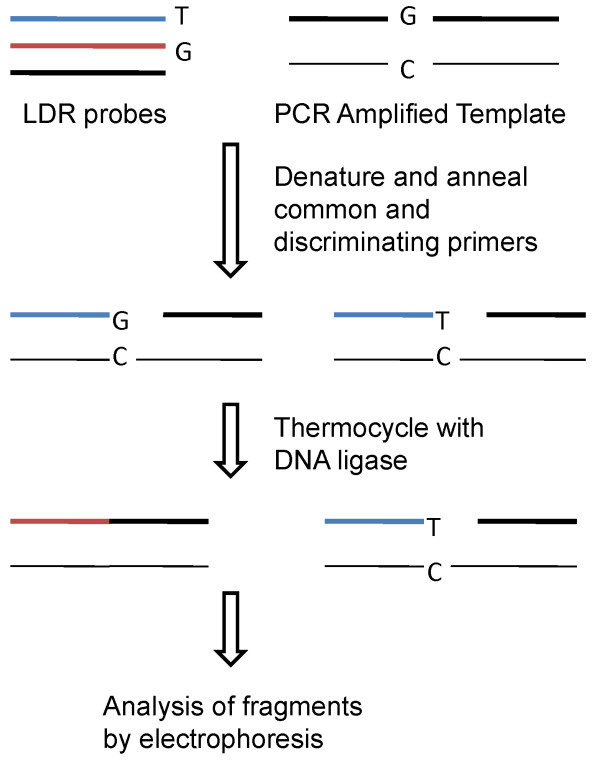
**Schematic representation of the ligase detection reaction**. PCR products are denatured and common (black) and discriminating primers (blue and red) annealed. DNA ligase only ligates those duplexes which contain exact matches and thermocycling with DNA ligase amplifies the ligated products.

Rondini et al. developed a sensitive assay for the detection of both lineages of WNV by coupling multiplex RT-PCR and ligase detection reaction (LDR) [84]. Multiple PCR primers amplify three distinct regions of the WNV cDNA. Each PCR primer contains between one and three degenerate positions to accommodate minor sequence variation at the primer binding sites. Within each PCR amplicon, LDR primer pairs are designed to identify SNPs (Figure 3). Ligation of the appropriate LDR primers results in fluorescently labeled products of different lengths that are then separated using capillary electrophoresis. The broad strain coverage was confirmed by testing 34 WNV isolates belonging to lineages 1 and 2. The detection limit was very low reaching 0.005 and 0.017 PFU for respectively the one-step or two-step procedure. Detection of LDR products could also be achieved by hybridization to a universal DNA microarray.

PCR based diagnostic assay requires specialized equipment and well-trained personnel which is difficult to obtain in developing countries. A relatively inexpensive assay for WNV is the loop-mediated isothermal amplification (LAMP) assay described by Parida et al. [86]. The LAMP assay is based on the principle of autocycling strand displacement DNA synthesis performed by Bst (Bacillus stearothermophilus) DNA polymerase and a set of two inner primers and two outer primers. The advantage of LAMP is that the amplification reaction can be performed under isothermal conditions between 63°C and 65°C, thereby obviating the need for a thermal cycler [89]. The RT-LAMP assay of Parida et al. [86] demonstrated a high degree of specificity for WNV and was more sensitive than the traditional RT-PCR detecting 0.1 PFU of WNV.

Another technology that works at isothermal conditions is the nucleic acid sequence-based amplification (NASBA) assay. Lanciotti et al. developed two NASBA assays each with a different detection format, i.e. electrochemiluminescence or 6-carboxyfluorescein-labeled virus-specific molecular beacon probes, for the detection of WNV and Saint Louis encephalitis (SLE) [87]. With these newly developed NASBA assays a diagnosis can be made within a hour and their sensitivity is similar or even greater than the sensitivity of their previously developed TaqMan assay.

A major disadvantage of all real-time PCR techniques is that new emerging WNV strains may acquire mutations in the PCR-primer binding sites, which render them undetectable to currently existing assays. In addition, new WNV isolates belonging to WNV lineage 2 may pose a problem. Indeed, data from an external quality assessment of the molecular diagnostic of West Nile virus showed that some of the participating laboratories could not detect lineage 2 viruses by their PCR assays [90]. To avoid that lineage 2 viruses are missed by the assay, Linke et al. developed a real-time PCR targeting a conserved region of lineage 1 and 2 WNV [91]. Eiden et al. developed two real-time quantitative RT-PCRs for the detection of lineages 1 and 2 WNV strains. The primers and probe were located either in the 5'-untranslated region or in NS2A. Both assays allowed detection of both lineages with high sensitivity [92]. Several groups started to combine detection and genotyping of WNV by real-time PCR. Zaayman et al. [85] performed genotyping of WNV strains by means of dissociation-curve analysis using the fluorescence resonance energy transfer probe technology whereas Papin et al. used a genome-wide, multiple primer-based real-time quantitative PCR assay [93]. Considerable efforts have been put into the development of a third generation of PCR, namely digital PCR (dPCR). It involves partitioning of a sample into thousands of nanoliter sized droplets by means of an emulsion droplet generator [94]. Subsequently, fluorescent labeled probes are added to the droplets and PCR is performed on a standard thermal cycler. After the PCR the droplets are analyzed by passing them in a single file in front of fluorescence detectors. Subsequently, the outcome of the reaction is determined using a rigorous statistical analysis. In a multiplex PCR, the reaction mixture contains varying concentrations of the different fluorogenic probes of the same color. It is then possible to identify the different probes on the basis of fluorescence intensity. Life Technologies have successfully applied their dPCR technology for the detection of WNV.

### 5.2. Serologic diagnosis of WNV infections

Following exposure to WNV, both IgM and IgG antibodies are produced. In most cases, IgM antibodies can be detected within 4 to 7 days after the initial exposure and may persist more than one year [95]. In comparison, anti-WNV IgG are reliably detected ~ 8 days after the onset of symptoms and they have a limited use in the initial diagnosis of WNV infection [71]. Flavivirus-infected sera show cross-reactions in serodiagnosis with heterologous flavivirus infections. Therefore, the plaque reduction neutralization test (PRNT) is still used as the reference assay for specific diagnosis of WNV infection. However, PRNT is a laborious test and must be carried out in a biosafety level 3 (BSL-3) facility as viable WNV viruses are used in this assay. For high-throughput screening, different ELISA methods (e.g. indirect IgG, IgM antibody-capture and blocking ELISA) have been developed over the last years [74]. ELISAs have the advantage of being rapid, reproducible and less expensive than other methods.

The WNV blocking ELISA measures the ability of antibodies present in sera to block the binding of a monoclonal antibody (mAb) to the NS1 protein [96-98], the E-protein [96] or WNV-specific antigens present in cell extracts of WNV infected cells [99]. The advantage of the method is that it is species independent as demonstrated by Blivitch et al. [96,99-101]. Their blocking ELISA based on the E-protein reliably detected flavivirus antibodies in several species of domestic mammals including horses, cows, pigs and cats [100]. Sotelo et al. developed a blocking ELISA with a monoclonal antibody recognizing domain III of the E glycoprotein [102]. After experimental infection of partridges this blocking-ELISA detected WNV-specific antibodies as early as 3 days post-infection whereas neutralizing antibodies were detected by PRNT at day 10. The diagnostic sensitivity was 100% compared to PRNT but the specificity was only 79.5%. Kitai et al. established an epitope-blocking ELISA based on NS1 that differentiated WNV from JEV infections in horse sera [98]. Since several vaccines under development are based on the prM and E proteins, NS1 based ELISAs will still be able to discriminate between vaccinated and naturally infected animals. While comparing different assays, they observed that neutralizing antibodies were detectable on day 7 by PRNT, and anti-WNV antibodies were observed from day 10 by conventional ELISA and day 12 in their blocking ELISA.

Indirect IgG and IgM antibody-capture ELISA (MAC) are used primarily for serological detection of WNV in acute or convalescent serum or CSF samples. However, there are limitations to these tests such as the lack of certain anti-species secondary antibody conjugates and cross-reactivity with other flaviviruses. Alonso-Padilla et al. described an indirect ELISA for detection of anti-WNV IgG antibodies based on recombinant insect-cell derived soluble WNV-E protein [103]. Comparison of this ELISA based on recombinant E protein with the ELISA using inactivated whole virus as antigen showed an equivalence in sera reactivity, with excellent specificity and sensitivity when compared with the "gold-standard" PRNT technique.

In an attempt to differentiate between St. Louis encephalitis virus (SLEV) and WNV, Chang et al. performed extensive mutagenesis within the cross-reactive epitopes of the E proteins of the two flaviviruses. Subsequently, each mutant E protein was presented on the surface of virus-like particles (VLP) to evaluate their diagnostic potential. The assay was validated using human serum samples from patients infected with SLEV, WNV or other flaviviruses. It was found that in the MAC-ELISA higher specificity was obtained using the VLP containing the mutant antigens [104].

To further improve the specificity of the WNV ELISAs, fragments of WNV proteins have also been used to differentiate between different flaviviruses. The DIII of the E protein is an immunoglobulin-like domain protruding from the otherwise smooth particle surface. Studies on the DIII of the E protein from JEV, DENV and WNV showed slight differences in their structures, particularly in areas that constitute virus neutralizing epitopes [105]. This observation led different groups to study the diagnostic potential of domain III of the E protein. Beasley et al. demonstrated that an ELISA with WNV-DIII produced in bacteria could differentiate clearly between antibody responses to WNV and those produced by other related flaviviruses, such as SLEV, JEV and Murray Valley encephalitis virus (MVEV) [106].

**Figure 4:**
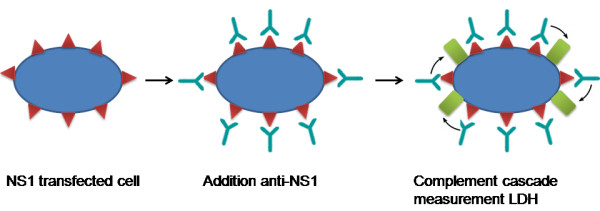
**CDC assay as developed by Kitai**. See text above for further details.

Other serological tests have been explored for use in diagnosis of WNV infections. Kitai et al. developed a complement-dependent cytotoxicity (CDC) assay (Figure 4) to measure antibodies to the West Nile virus NS1 protein in horses [107]. The antigen used for the assay was obtained from a stably transfected cell line that constitutively expressed the NS1 protein of the WNV Eg101 strain. After incubation of the cells with heat-inactivated test serum, commercial rabbit complement is added and release of lactose dehydrogenase from cells was measured. A comparison between a conventional ELISA, the blocking ELISA, and a virus neutralization test (VNT) revealed that this system detected anti-NS1 antibodies at similar time points as the conventional ELISA, but later as the VNT and earlier as the blocking ELISA.

Microsphere-based immunoassays (MIAs) are becoming increasingly popular for the diagnosis of many diseases. This technology is based on the covalent bonding of antigen to microspheres. The detecting device is a simplified flow cytometer and the procedure can be completed in a few hours. Balasuriya et al. developed four MIAs using recombinant WNV E, NS1, NS3 or NS5 proteins for the detection of equine IgG antibodies in sera of vaccinated or naturally infected horses [108]. The NS-based MIAs were less sensitive than both PRNT and E-MIA. However, the NS1-MIA was able to distinguish between horses vaccinated with the canarypox virus vaccine and horses that were naturally infected. Johnson et al. produced a human IgM specific WNV/SLE MIA using recombinant WNV prM and E and SLEV antigen extracted from mice brains as detecting antigens [109].

A surface enhanced Raman scattering (SERS) immunoassay for antibody detection in serum was developed by Neng et al. [110]. This assay utilizes gold nanoparticles coated with the E protein of WNV as the SERS-active substrate and protein A/G conjugated with the Raman tag malachite green (MG) as a bi-functional Raman tag/antibody binding reporter. The assay was validated by incubation of the E protein-coated gold nanoparticles with immune serum from rabbits. Subsequent laser interrogation of the sandwiched immunocomplex revealed a SERS signaling response diagnostic for MG. Since protein A/G can interact with a range of mammalian antibody subclasses, this SERS immunoassay can be used for different mammalian species (but not avian due to low binding affinity for protein A/G) without the requirement of species specific secondary antibody conjugates.

### 5.3. WNV antigen detection

The VecTest^® ^is an antigen panel assay designed by Medical Analysis Systems to detect WNV, SLE and Eastern Equine Encephalitis (EEE). It uses a detection dipstick coated with specific antibodies. Although it is less sensitive than the plaque assay in Vero cells or RT-PCR [111], it has the advantage that it gives a result in less than 20 minutes and it does not require sophisticated equipment.

Two groups developed sensitive antigen capture ELISAs (ACE) for the detection of secreted NS1. MacDonald et al. produced two ACEs for the detection of NS1 in experimentally infected hamsters [112]. In their first ACE a polyclonal antiserum was used as detecting antibody, and in their second ACE they used the same monoclonal antibody for capturing and detecting the NS1 antigen. The first ACE was more specific for recombinant forms of NS1, while the latter detected native NS1 at high sensitivity. The detection limit was less than 1 ng/mL. Chung et al. developed a similar ACE using two different monoclonals [113]. Their ACE detected as little as 0.5 ng/mL of soluble NS1 and showed no cross-reactivity with yellow fever, Dengue and SLE virus NS1.

A membrane-based electrochemical nanobiosensor that recognizes viral particles or virus E protein was fabricated by Nguyen et al. by putting a nanoporous alumina membrane over a sensing electrode [114]. IgM raised against domain III of protein E was used as the specific biorecognition probe for WNV particles. This assay was highly sensitive toward whole WNV particles with a detection limit of 2 viral particles per 100 mL, which is comparable to PCR techniques. This might be useful for the detection of virus early during infection of the host, i.e. before the onset of antibody production.

## 6. Vaccination

A review of the trends in vaccine development was published in 2007 by Dauphin et al. [74]. The current paper focuses more on the evolution in vaccine development since 2007.

### 6.1. Licensed West Nile virus vaccines for animals

Although no human vaccine is available to date, there are currently three WNV vaccines licensed for horses (Table 2). The first licensed vaccine was developed by Fort Dodge Animal Health, which is now subsidiary of Pfizer. It contains a formalin-inactivated, whole West Nile virus. This vaccine is currently commercialized in the USA under the trade name West Nile-Innovator^® ^and is quite effective. Indeed, 12 months after two doses of West Nile-Innovator^® ^94% of the animals were protected against viremia after challenge. In a safety trial of the vaccine, less than 5% of the horses showed adverse responses to vaccination [115]. Another killed virus vaccine (Vetera^® ^WNV vaccine) developed by Boehringer Ingelheim Vetmedica was also licensed by the United States Department of Agriculture (USDA). A third commercialized WNV vaccine in the United States for horses is Recombitek^® ^Equine West Nile Virus Vaccine (Merial, now Sanofi Aventis), which is a chimeric recombinant canarypoxvirus vaccine [116]. This vaccine expresses the prM and E genes derived from a 1999 New York isolate of West Nile virus (WNV). All of the vaccinated horses developed neutralizing antibodies against WNV and showed significantly fewer clinical signs of WNV disease upon challenge [117]. Also an inactivated form of this chimeric vaccine has been licensed by the USDA. In 2005, a WNV DNA plasmid-based vaccine was licensed in the United States by Fort Dodge Animal Health/Pfizer under the trade name of West Nile-Innovator^® ^DNA. The vaccine contains an unformulated plasmid DNA encoding the prM and E protein of WNV and MetaStim™ as adjuvant. The vaccine is administered intramuscularly and a second dose is given two to four weeks after the first. This vaccine has recently been discontinued by Pfizer. A *Flavivirus *chimera vaccine for horses (PreveNile, Intervet) containing West Nile virus pre-membrane (prM) and envelope (E) genes (from the NY99 strain) in a backbone of yellow fever (YF17D vaccine virus), was granted a full license by USDA in 2006. However, in 2010 it was recalled from the market after the observation of adverse effects like acute anaphylaxis, colics, respiratory distress and even death in horses.

**Table 2:** Overview of the different commercialized and candidate West Nile vaccines.

Name	Viral antigen(s)	State of development	Reference
West Nile-Innovator (Pfizer) RecombiTek (Merial)	Whole virus WNV prM-E in canarypox virus	Commercialized for horses Commercialized for horses	[63]

West Nile-Innovator DNA	Plasmid DNA prM/E	Licensed for horses	[23]

PreveNile (Intervet)	WNV prM-E in yellow fever backbone	Commercialized for horses (recalled in 2010)	

Vetera West Nile vaccine (Boehringer Ingelheim)	Killed virus	Commercialized for horses	

ChimeriVax (Sanofi)	Yellow fever PrM-E substituted by WNV prM-E	Phase II human clinical trial	[5,8]

WN-DEN4	WNV prM-E in dengue-4 backbone	Phase II human clinical trial	[72]

VRC303 (NIAID/Vical)	Plasmid encoding WNV prM and E	Phase I human clinical trial	[41]

STF2Δ.EIII	S. typhimurium flagellin fused to E domain III	Evaluated in mice	[55]

rWNV-E_T_	Truncated protein E	Evaluated in mice and horses	[19,42]

SRIP	prM-E VLPs	Evaluated in mice and horses	[12]

RepliVAX WN	Single-cycle West Nile virus	Evaluated in mice [118], hamsters [119], non-human primates [120]	

	Plasmid encoding E domain III fused to P28	Evaluated in mice	[22]

DIII-C-AP205	E domain III coupled to bacteriophage AP205	Evaluated in mice	[87]

FLU-NA-DIII	E domain III inserted into NA of influenza	Evaluated in mice	[54]

CAdVax-WNVII	C, preM, E and NS1 expressed in adenovirus	Evaluated in mice	[81]

### 6.2. WNV vaccines under development

A recombinant influenza virus expressing domain III of the WNV E protein has been evaluated as a WNV vaccine candidate in a mouse model [121]. The WNV DIII was cloned in the N-terminal region of the influenza virus neuraminidase destroying the functional activity of the influenza protein. Subcutaneous immunization of mice with the vaccine, FLU-NA-DIII, resulted in higher virus-neutralizing and WNV-specific IgG ELISA titers than intranasal administration. In addition, cellular DIII-specific responses as determined by IFN-γ ELISPOT assay were also stronger in the subcutaneously immunized group. After subcutaneous challenge with WNV, higher morbidity as assessed by loss of body weight was observed in the intranasal immunized group. Survival rates were 100% and 75% in mice immunized with FLU-NA-DIII via the subcutaneous or intranasal route, respectively.

Schepp-Berglind et al. created an adenoviral vaccine vector (CAdVax-WNVII) expressing four WNV proteins, C, prM, E and NS1. Although these proteins originated from a lineage II virus strain, serum samples collected after vaccination of mice with CAdVax-WNVII contained antibodies that neutralized lineage I and II viruses. In vaccinated mice T cell activity against WNV antigens was observed in splenocytes after re-stimulation in vitro with WNV infected target cells [122].

The emergence of pathogenic lineage 2 strains in Europe raised the question whether the existing WNV vaccines, mainly based on lineage 1 strains, can also protect against the new circulating lineage 2 strains of WNV. Minke et al. demonstrated that Recombitek^® ^Equine West Nile, that expresses the prM/E genes of lineage 1 strain in a recombinant canarypox virus, could protect horses against a contemporary neurovirulent lineage 2 WNV isolate [123]. Finally, Yamshchikov et al. reported that an attenuated non-epidemic West Nile virus strain of lineage 2 can be used as an effective vaccine against a virulent epidemic strain of lineage 1 in mice [124].

Another strategy that has been evaluated is vaccination with purified viral proteins. Although these vaccines protect against disease in animal models, multiple injections and/or strong adjuvants were required to reach acceptable efficacy. Demento et al. [125] formulated recombinant E protein onto poly(lactic-co-glycolic acid) (PLGA) nanoparticles that contained CpG oligonucleotides at their surface. Activation of dendritic cells as determined by the secretion of IL-6 and IL-12 was stronger when the CpG-modified E protein-loaded nanoparticles were used compared with E protein adsorbed to Alhydrogel. C3H/HeN mice immunized with encapsulated E protein in CpG modified nanoparticles or with E protein adsorbed to alhydrogel elicited equivalent titers of IgG however, the isotype profiles were very different. Only CpG-modified particles loaded with E protein raised high IgG2a and IgG2b titers. Lymphocytes from mice vaccinated with encapsulated E protein in CpG modified nanoparticles produced higher levels of IFN-γ and IL-2 in vitro after re-stimulation with E protein compare to lymphocytes from Alhydrogel-rWNV-E_T _immunized mice. CD8^+^CD44^+ ^T cells from mice vaccinated with CpG/rWNV E nanoparticles had a larger KLRG1^+^CD127^- ^population, a subset of terminally differentiated effector cells.

Martina et al. produced domain III protein of the E protein of WNV (rDIII) and compared it with a β-propiolactone (BPL) inactivated WNV vaccine [126]. Neutralizing antibodies against WNV were detected in all mice. Interestingly, cross-neutralizing IgG against JEV also were produced. Mice vaccinated with rDIII and challenged with either WNV or JEV were protected against morbidity as determined monitoring the body weight. However, the survival rates were lower (80% to WNV and 60% to JEV) compared to mice vaccinated with BPL inactivated WNV (100% to WNV and 80% to JEV). McDonald et al. fused bacterial flagellin to the domain III of the WNV envelope protein **(**STF2Δ.EIII) providing the fusion protein the ability to engage the TLR5 receptor. Mammalian hosts detect the conserved domain on flagellin monomers through TLR5, which triggers proinflammatory and adaptive immune responses. Mice injected either subcutaneously or intraperitoneally with the flagellin-DIII fusion protein produced significant levels of anti-WNV E IgG as determined by ELISA. In addition, sera from vaccinated mice had neutralizing antibody titers > 1/40. In a mouse model, > 90% survival was observed in animals that were immunized with STF2Δ.EIII [127]. Spohn et al. also have used recombinantly expressed domain III of the WNV E protein as an immunogen. This group chemically coupled the DIII protein to VLP derived from bacteriophage AP205. This conjugate vaccine DIII-C-AP205 was more immunogenic in mice than a mixture of corresponding amounts of free DIII and its carrier AP205. Neutralizing antibodies could be detected in 75% of the mice after one injection with the DIII-C-AP205 vaccine while all animals scored positive after three injections. The latter group was also fully protected against a lethal challenge with the virus [128].

Finally, a fourth strategy uses DNA vaccination as a platform for WNV vaccination. Davis et al. were the first to demonstrate that plasmid DNA encoding the WNV membrane (M) and envelope (E) proteins injected intramuscularly in mice and horses provided protection against a WNV challenge [129]. DNA vaccination resulted in both a humoral response as well as a strong Th1 response. This study paved the way for the licensing of the first DNA vaccine for animal use, i.e. West Nile-Innovator^® ^DNA. Later on, other administration routes and carrier mediated delivery of the WNV DNA vaccines have been exploited. Zhao et al. showed that inoculation of plasmid (mixed with colloidal gold) via intravenous and intradermal injection elicited stronger and more sustained neutralizing immune responses than intramuscular or intraperitoneal injection [130]. Prow et al. used a nanopatch with a microneedle array to deliver a West Nile virus DNA vaccine that was complexed to poly(ethylenimine) [131]. This simple needle-free technique resulted into an effective vaccine delivery with a cutaneous expression of encoded proteins within 24 h [131]. Dunn et al. evaluated DNA vaccines with derivatives of the WNV E gene (full length, truncated E or DIII region) conjugated to the P28 region of the complement protein C3d. Mice were vaccinated three times either intramuscularly or by the gene gun route. The latter resulted in higher IgG titers against WNV DIII. Gene gun DNA vaccination induced primarily a Th2 response (characterized by IgG1 antibodies) whereas intramuscular administration resulted more in a Th1 response (characterized by IgG2 antibodies). Eighty percent of the mice vaccinated by gene gun with DIII-DNA survived a lethal WNV challenge with little weight loss, while no mice survived in the intramuscularly vaccinated group. However, the survival rates after IM administration increased to 60% by conjugating P28 to DIII [132]. At the moment, all the commercially available DNA vaccines against any pathogen contain unformulated DNA. Chang et al. developed a plasmid DNA (pDNA) that after transfection gives rise to single-round infectious particles (SRIPs) based on WNV. Flavivirus RNAs that contain large deletions in the capsid gene cannot produce infectious virions but retain the ability to replicate their RNA backbone and express prM and E proteins. After transfection, the plasmid DNA generates two different mRNAs: one encoding capsid protein and the other for the prM, the E, non-structural proteins and a truncated capsid. Only the latter RNA molecule can replicate and become incorporated in the SRIPs. Cells transfected with SRIPs will produce subviral particles that contain the prM and E-proteins but lack virus genomic material. This plasmid DNA vaccine was delivered using a gene-gun with DNA-coated gold particles. Vaccination of mice with plasmid DNA encoding SRIPs elicited higher overall and neutralizing antibody titers than after vaccination with a plasmid encoding prM-E [133]. After intraperitoneal challenge, all mice vaccinated with SRIPs were protected against morbidity whereas some mice in the prM-E DNA plasmid group developed signs of disease although all mice survived infection. The SRIP vaccine also was able to induce virus-neutralizing antibodies in horses. A similar strategy was followed by Mason et al. They produced RepliVAX WN, a live-attenuated virus in which the gene encoding the capsid protein was deleted from the WNV genome. Vaccination with RepliVAX WN induced protective immunity in mice [118], hamsters [119] and non-human primates [120]. Nelson et al. characterized the nature of the immune response to RepliVAX in mice [134]. They found that the number of B cells secreting IgG specific for NS1 peaked at day 8 and was dose-dependent. Long-term presence of NS1-specific plasma cells through 8 months was observed. The IgG subclass of the induced antibodies was predominantly IgG2 while only little IgG1 was produced. Immunized mice mounted a strong CD8^+ ^T cell response against NS4B_2488 _and E_347 _that peaked at day 6. The cytotoxic activity of these cells was confirmed by analysis of the killing of peptide-pulsed target cells. The CD8^+ ^T cells produced TNF-α and IFN-γ after stimulation with the NS4B_2488 _peptide. Significant CD4^+ ^T cell responses were also detected against peptides NS3_1616_, E_641_, E_431 _and NS3_2066 _that peaked on day 13. Specific cytolysis of NS3_2066 _pulsed cells by CD4^+ ^splenocytes was observed by day 6 and peaked on day 8. CD4^+ ^T cells produced predominantly IFN-γ and no IL-4 following restimulation with peptide in vitro. Memory CD4^+ ^and CD8^+ ^T cells were detected 8 months post immunization [134].

### 6.3. Clinical trials with West Nile virus vaccines in humans

At present, there are no FDA-approved vaccines for human use but several clinical trials are ongoing. In 2005, Acambis (Sanofi-Pasteur) successfully completed a Phase I clinical trial with its live-attenuated ChimeriVax-WN. ChimeriVax-West Nile (Acambis, Sanofi-Pasteur) utilizes the attenuated YFV vaccine strain (17D) to build a live chimeric virus that consists of the prM and E proteins of WNV in the context of the YFV capsid and non-structural proteins [135]. ChimeriVax-West Nile is the most advanced vaccine in development. In the first part of a Phase II trial in healthy adults 18-40 years of age, a single dose of ChimeriVax-West Nile raised neutralizing antibodies 28 days after vaccination [135]. The second part of the Phase II trial determined safety and tolerance in healthy individuals over 41 years of age. Seroconversion was achieved at day 28 by more than 96% of the healthy adults in both age groups. Another chimeric vaccine (WN-DEN4) that uses attenuated dengue virus as a backbone for prM-E genes of WNV [136] is being evaluated in a Phase II human trial at the John Hopkins School of Public Health in adults 18-50 years of age. The Vaccine Research Center (VRC) at the National Institute of Allergy and Infectious Diseases (NIAID) has, in collaboration with Vical, developed a DNA plasmid-based vaccine. In 2005, the VRC initiated a successful Phase I clinical trial demonstrating its safety, tolerability and ability to induce neutralizing antibodies. Subsequently, a second-generation DNA vaccine using an improved vector was evaluated in a Phase I clinical trial. Naked plasmid DNA was administered via needle-free intramuscular injection on days 0, 28 and 56 with at least 21 days between injections. The plasmid in this vaccine is incapable of replicating in animal cells and does generate infectious virions. The vaccine was well tolerated without serious adverse events. All individuals that completed the 3-dose vaccination schedule developed neutralizing antibodies [137]. The majority of the subjects developed a CD4^+ ^response rather than a CD8^+ ^response as assessed by intracellular cytokine staining. Vaccine-induced T cell responses were mainly directed against WNV E protein.

## 7. Conclusions

West Nile virus remains a serious threat to the public health, especially to very young, elderly and immunocompromised individuals. There is currently no antiviral treatment to cure WNV infections and only supportive care can be administered. Ribavirin [138], interferon-α [57,139] and WNV-specific immunoglobulin [56,140] have all been considered as specific treatments for WNV disease, but no rigorously conducted clinical trials have been completed. Diagnostic tests have improved considerably and allow a rapid detection of the presence of WNV. Vaccine development against WNV continues to progress. Four WNV vaccines are currently available in the USA for horses. Although several clinical trials in various phases in humans are ongoing, it will take several years before any vaccine is available. Given the relatively low incidence of WNV neuroinvasive disease in healthy individuals and the focal occurrence of WNV epidemics thus far, vaccination will likely target the groups at higher risk for WNV neuroinvasive infection. At the moment, the best way to prevent West Nile virus infection remains to avoid mosquito bites.

## 8. Competing interests

The authors declare that they have no competing interests.

## 9. Authors' contributions

MDF developed the structural design of the review, organized the work and did together with NS the drafting of the manuscript. SU and MD performed detailed and critical revisions of the manuscript. All authors read and approved the final manuscript.
